# Design and construction of a low-cost nose poke system for rodents

**DOI:** 10.1016/j.mex.2016.04.002

**Published:** 2016-04-19

**Authors:** Giorgio Rizzi, Meredith E. Lodge, Kelly R. Tan

**Affiliations:** Biozentrum, University of Basel, Klingelbergstrasse 50/70, 4056 Basel, Switzerland

**Keywords:** Arduino based nose poke system for rodents, Operant behavior, Rodent, Reward, Nose poke, Optogenetics, Lowcost

## Abstract

Operant behavioral tasks for animals have long been used to probe the function of multiple brain regions (i.e., understanding the role of dopamine in electrical brain stimulation reward [1], or determining the rewarding properties of feeding oriented brain pathways [2]). The recent development of tools and techniques has opened the door to refine the answer to these same questions with a much higher degree of specificity and accuracy, both in biological and spatial-temporal terms [3], [4]. A variety of systems designed to test operant behavior are now commercially available, but have prohibitive costs. Here, we provide a low-cost alternative to a nose poke system for mice. Adapting a freely available sketch for ARDUINO boards, in combination with an in-house built PVC box and inexpensive electronic material we constructed a four-port nose poke system that detects and counts port entries. To verify the applicability and validity of our system we tested the behavior of DAT-CRE transgenic mice injected with an adeno-associated virus to express ChannelRhodopsin 2 in the Ventral tegmental area (VTA) and used the BNC output to drive a blue laser coupled to a fiber implanted above the VTA. Over 6 days, mice perform as it has been reported previously [5] exhibiting a remarkable preference for the port that triggers optogenetic stimulation of VTA dopamine neurons.

• We provide a low cost alternative to commercially available nose poke system.
• Our custom made apparatus is open source and TTL compatible.
• We validate our system with optogenetic self-stimulation of dopamine neurons.

## Method

Operant behavioral tasks designed for rodents are often used to probe the involvement and function of different brain regions linked to a variety of physiological and pathological brain states. The systems used to perform these experiments are often very expensive and beyond the reach of small labs or low budget projects.

Here we recapitulate the steps taken to construct a custom-made nose poke system designed for mice, taking advantage of open source technology and inexpensive materials.

Arduino boards are becoming increasingly popular due to their versatility and low cost. They have dedicated software and hardware that allow them to be programmed and therefore be applied to suit any need.

Starting from a freely available sketch (Supl. Ref. 1) containing source code designed to make a laser tripwire, we applied the same principle to detect the nose entries to a port and built a nose poke system around it.

The circuit is composed of a light dependent resistor (LDR) that gets exposed to a light source, in our case a red LED, a second LED (here green) that turns on every time the light beam onto the LDR is interrupted and an output BNC connector that drives the laser (GMP S.A. MBL-F-473-500 mW) for optogenetic stimulation.

For a list of all the necessary electronic components to build the Arduino based four-port nose poke system see Table 1.

Our system includes four different ports that have separate circuits connected in parallel. The ARDUINO UNO board processes all of the inputs and outputs.

**Fig. 1:**
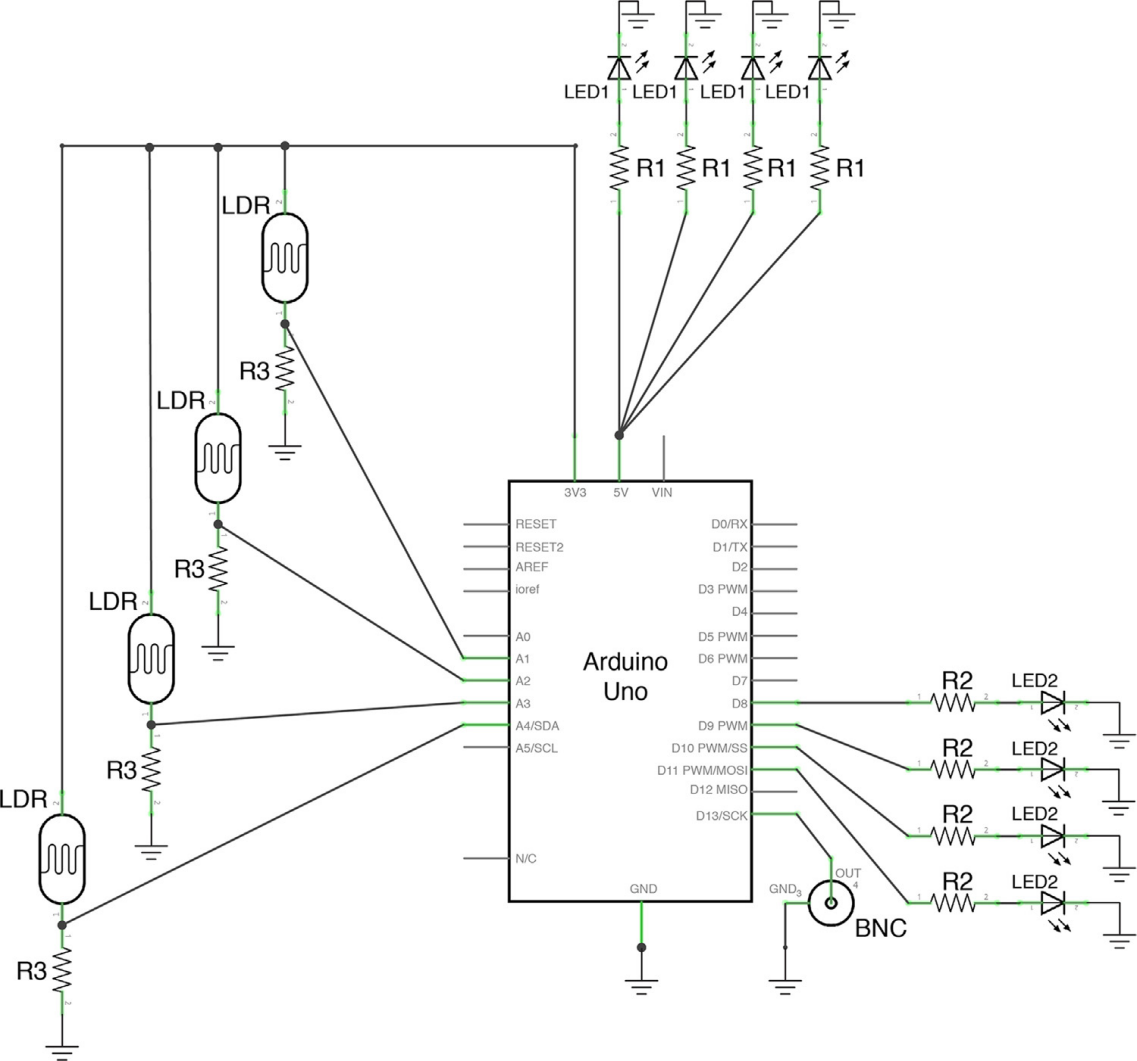
Electronic circuit schematic of the nose poke system. Schematic diagram of the circuitry needed for the custom-made nose poke system depicting positioning and connection of each component. The names used in this schematic are reported in Table 1 for the correct selection of each component.

As schematized in Fig. 1, the red LEDs (LED1) used as light sources for the LDRs are each connected in series with a 100 Ω resistor (R1) and all connected to the 5 V port of the Arduino board and the ground port, creating a very simple circuit to provide power to the LED1. The light sensing circuits are composed of an LDR connected between the 3.3 V port of the Arduino board and the ANALOG-IN ports A1 to A4. A voltage divider is created connecting a 10 kΩ resistor (R3) between the LDRs and ground (Fig. 1).

The green LED (LED2) circuits are formed connecting a 120 Ω resistor (R2) between DIGITAL ports D8 to D11 and the LED2s, which then are connected to ground (Fig. 1).

Finally the DIGITAL port D13 is connected to the live contact of the BNC connector and the shielding to ground (Fig. 1).

**Fig. 2:**
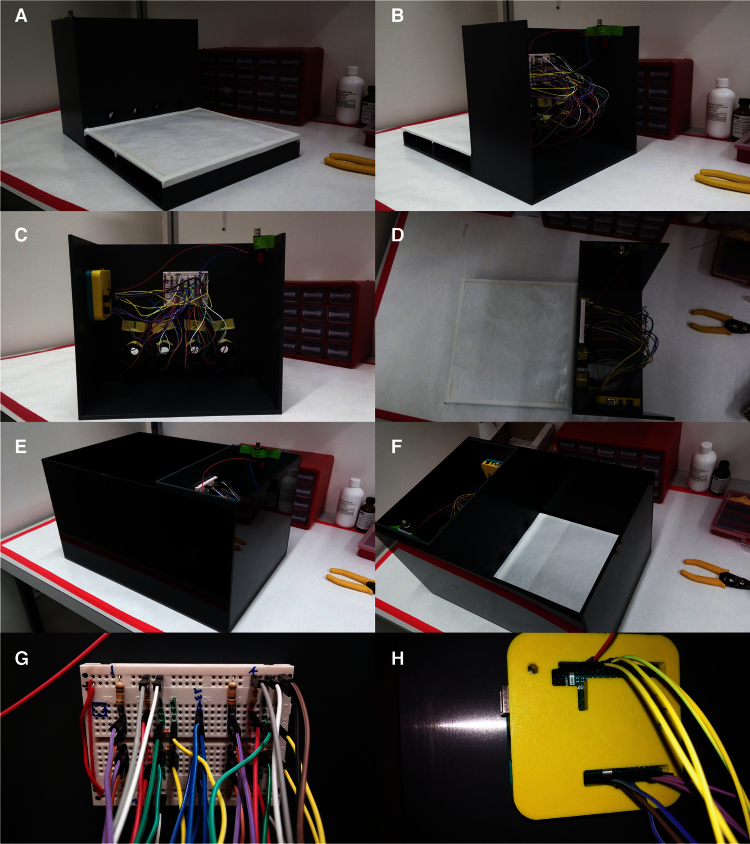
Illustrating images of the nose poke system. Images of the frame and electronic components assembled and ready to use. (A) Front view. (B) Rear view. (C) Back view. (D) Top view. (E) Rear view of the frame placed inside an open field box to prevent the mice from falling off the platform. (F) Top view of the frame placed inside open field. (G) Close view of the breadboard used to interface the connections between electric components and the Arduino board. (H) 3D printed case housing the Arduino board.

We designed the frame that would hold all the electronic components to fit a rectangular PVC box normally used for open field experiments with mice (Fig. 2E and F). We developed our design using SketchUp, a freely available and intuitive 3D modeling software (see Supl. Fig. 1). The frame is composed of two semi-closed compartments one designed to hold the mice while they perform the tasks and the other designed to hold all electronic components and connections (Fig. 2A and B). The front compartment has only a platform and a wall with four 15 mm diameter holes (nose poke ports) equally spaced and with the lower edge 1 cm from the floor platform, each having a 5 mm diameter hole above that holds the green LED2s.

On the back compartment, surrounding each nose port, there is a structure with 5 mm holes directly above and below the port designed to be aligned vertically and hold the red LED1 above and the LDR below. Thus, when the mouse snout gets into the port it creates a shadow over the LDR changing the value of the resistance (Supl. Fig. 1).

To facilitate the assembly of the electronic components onto the frame we used a breadboard to hold all resistors and used a 3D-printed case that holds the Arduino board (Fig. 2C, G and H). The SketchUp design was then implemented and constructed by the in-house workshop using PVC material. All the detailed measurements of our design can be found in the (Supl. File 1).

Once all the components were placed in the frame, the code (Supl. File 2) could be loaded on the Arduino board allowing the system to be used. For a detailed guide on how to upload sketches on Arduino boards see Supl. Ref. 2.

The Arduino board must be connected to a computer in order to communicate the nose pokes. Power can be supplied to the Arduino Uno either via USB cable or an external power adapter. To initialize an experiment once the board is connected to the PC, the Arduino IDE needs to be launched. Once the serial port monitor window is opened, the board is reset and starts executing the code. The first header line will be transmitted through the serial port, listing the port number, poke number and a time stamp. From that point on, every time there is a nose poke, its information will be transmitted as a new row of text with comma separated values. At the end of the session the list must be manually copied to a text file, which will then be saved in a comma separated values format. Closing and opening the serial monitor window of the Arduino IDE will reset the board and prepare it for a new session.

The serial port can be also monitored through third party software and/or a secondary code can be implemented to automatically copy and store the data transmitted by the Arduino board as an alternative to the manual process.

**Fig. 3:**
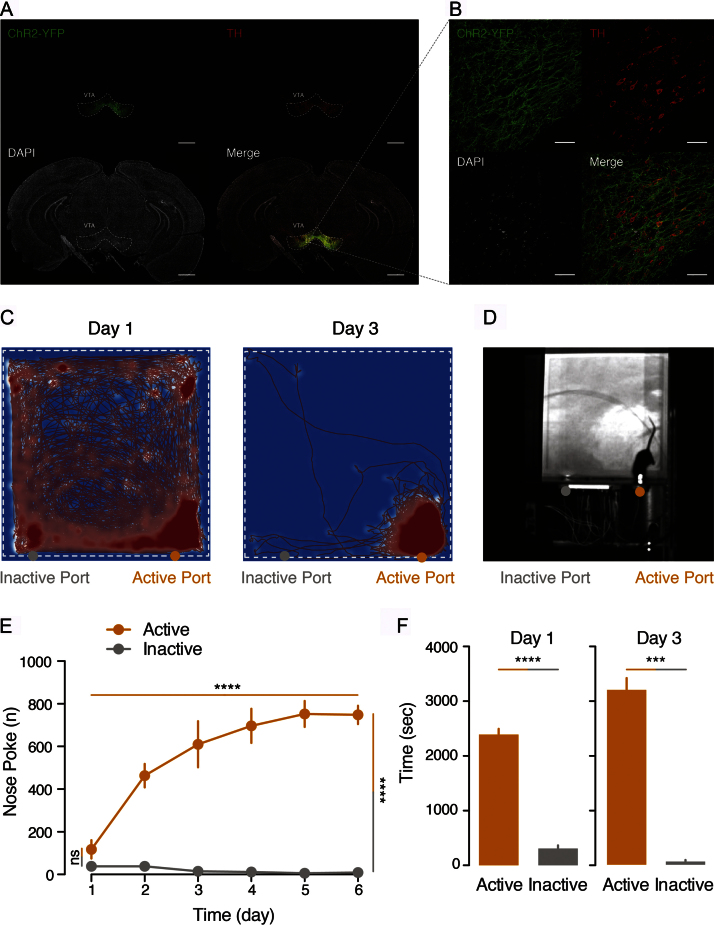
Quantification of dopamine neuron optogenetic self-stimulation. Validation of the system through dopamine self-stimulation in DAT-CRE transgenic mice. A) Infection site and TH counter stain verifying infection and dopamine selectivity of ChR2-YFP. Scale bar 1 mm. B) Magnified view of the VTA area showing overlap of TH staining and ChR2-YFP expression. Scale bar 100 μm. C) Example track plot of the first and third sessions of a single mouse overlaid with a normalized heatmap illustrating the distribution of time spent on the platform. D) Example image of a mouse nose poking in the active port. E) Quantification of the nose pokes in the active and inactive ports over time (two-way Anova repeated measures, effect across days F(5,20) = 19.76; p < 0.0001, effect between ports F(1,4) = 115.98; p = 0.0004). F) Quantification of the time spent near the active and inactive ports on the first day (2392 ± 102.3SEM and 308 ± 55.87SEM; p < 0.0001 *t*-test) and the third day (3205 ± 216.5SEM and 59 ± 29.74SEM; p = 0.0001 *t*-test).

To test the functionality of our system we decided to use DAT-CRE transgenic mice (Jackson Laboratory stock number: 006660) injected with DIO-ChR2-EYFP in the VTA and implanted with an optic fiber [6] above this region to deliver light and activate dopamine neurons. For this specific experiment the two central ports of the frame were blocked so that mice could only nose poke in either port most lateral to the frame (Fig. 3). The Arduino code was modified to make one of the ports inactive, the nose pokes were quantified but no light delivery was triggered following an entry to the inactive port. In response to an entry to the active port the BNC output connector triggered a 3 s light delivery at 50 Hz with pulse duration of 5 ms. During the 3 s light simulation no nose pokes were quantified.

Six to eight weeks old DAT-CRE transgenic mice (n = 3) underwent stereotactical surgery during which 300nl of AAV5-EF1α-DIO-ChR2-H134R-EYFP bilaterally with the following coordinates from bregma (−3.4 mm AP; 0.45 mm ML; 4.35 mm DV). During the same surgery a single optic fiber implant was placed with a 10° angle above the VTA to deliver light stimulation.

Three weeks after the surgery all animals were placed in the nose poke system for a daily 60 min session for a total of six days. The behavior of the animals during each session was recorded and tracked (Fig. 3D, Supl. Video 1), using AnyMaze software. The time spent nearby the active and inactive port was quantified automatically and the number of nose pokes in each port was counted and monitored by the Arduino board through the serial port monitor of its dedicated software.

As expected the animals displayed a very strong preference for the active port having an average number of nose pokes of 37.33 ± 11.33SEM in the inactive port and 116.7 ± 43.10SEM in the active port on day 1 and 8 ± 4SEM in the inactive port and 746 ± 42.53SEM in the active port on day 6 (Fig. 3E, effect between ports F(1,4) = 115.98; p = 0.0004 two-way Anova repeated measures,).

As displayed in Fig. 3C and F already on the third day of testing there was a significant preference for the active port (day 1 p < 0.0001 and day 3 p = 0.0001 *t*-tests) in all the animals confirming the validity of the preparation and the nose poke system. After the sixth day of testing all animals were intracardially perfused and fixed, the brains were slices and the infection and optic fiber placement were verified (Fig. 3A and B). Confocal images of the brains confirmed the restriction of the ChR2 infection to the VTA area and immunohistochemistry counterstaining with tyrosine hydroxylase (TH) confirmed the selectivity of ChR2 expression in dopamine neurons.

Here we provide a recapitulation of the making and testing of a custom-made Arduino-based nose poke system that allows for operant behavioral testing in mice at a fraction of the cost of commercially available systems. Due to the versatility of its microcontroller, this system can easily be modified to modulate any external device that is TTL compatible, and adapted to other types of operant switches such as lever presses or pressure plates.

## Figures and Tables

**Table 1:** Inventory. List of all the electronic materials needed to build a four-port nose poke system designed for mice.

Item	Quantity	Item Number	Company	Schematic Name
Arduino Uno	1	642818	Distrelec	Arduino Uno
100 Ω Resistor	4	722324	Distrelec	R1
120 Ω Resistor	4	728023	Distrelec	R2
10 kΩ Resistor	4	728104	Distrelec	R3
Red LED	4	632048	Distrelec	LED1
Green LED	4	632043	Distrelec	LED2
LDR	4	631603	Distrelec	LDR
BNC Connector	1	103032	Distrelec	BNC
Breadboard	1	920250	Distrelec	N/A
Jumper Cables	50–100	1705	Pololu	N/A

## Appendix A Supplementary data

Fig. S1 Example views of SketchUp design and software. Images illustrating the design of the frame in SketchUp showing rear view, front view and close ups. All dimensions are available in the Supplementary File 1.

Supplementary File 1 SketchUp file with design.

Supplementary File 2 Arduino file with the code.

Video 1 Video of animal nose poking for self-stimulation.

Table S1 Inventory including prices. List of all the electronic materials needed to build a four-port nose poke system designed for mice, including the prices for the components bought in Switzerland.
