# Supplementary figures and images for: Design and construction of a low-cost nose poke system for rodents
Source: MethodsX. 2016 Apr 19;3:326–32. doi: 10.1016/j.mex.2016.04.002 (PMC4855068; doi:10.1016/j.mex.2016.04.002)

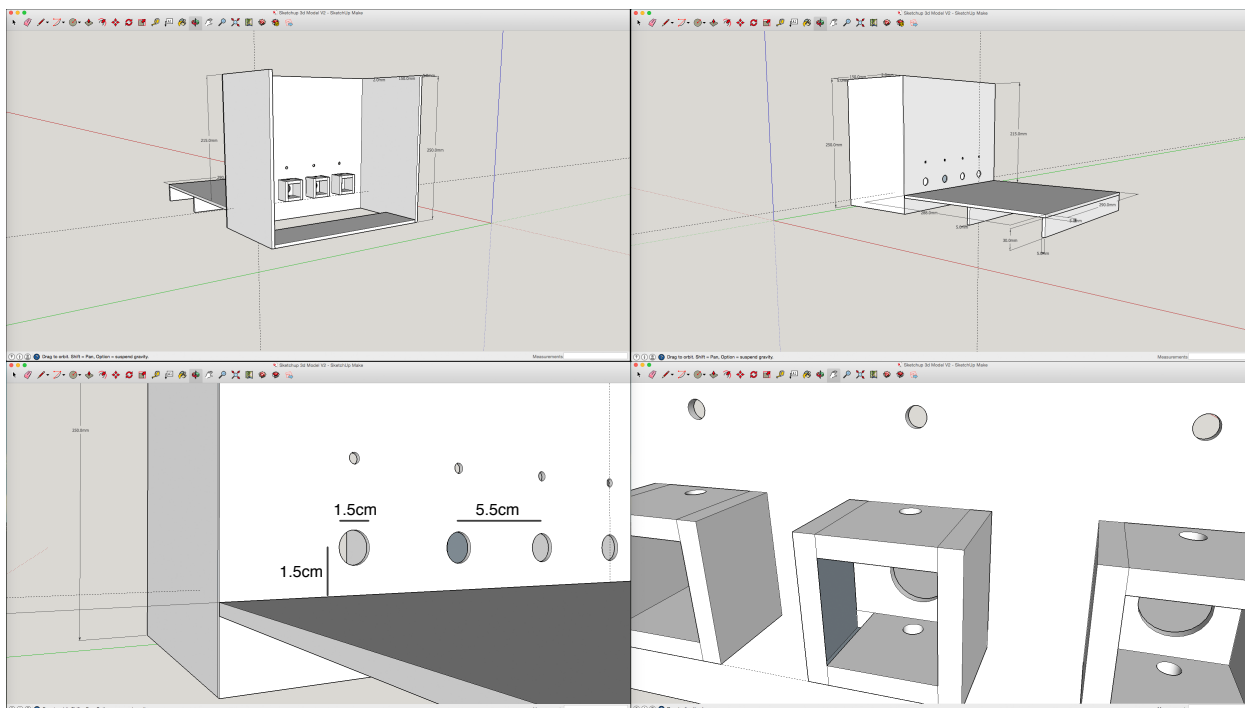

Rizzi et al. Supplementary Figure 1.

Supplement: Fig. S1 — Example views of SketchUp design and software. Images illustrating the design of the frame in SketchUp showing rear view, front view and close ups. All dimensions are available in the Supplementary File 1. [file mmc1.pdf]

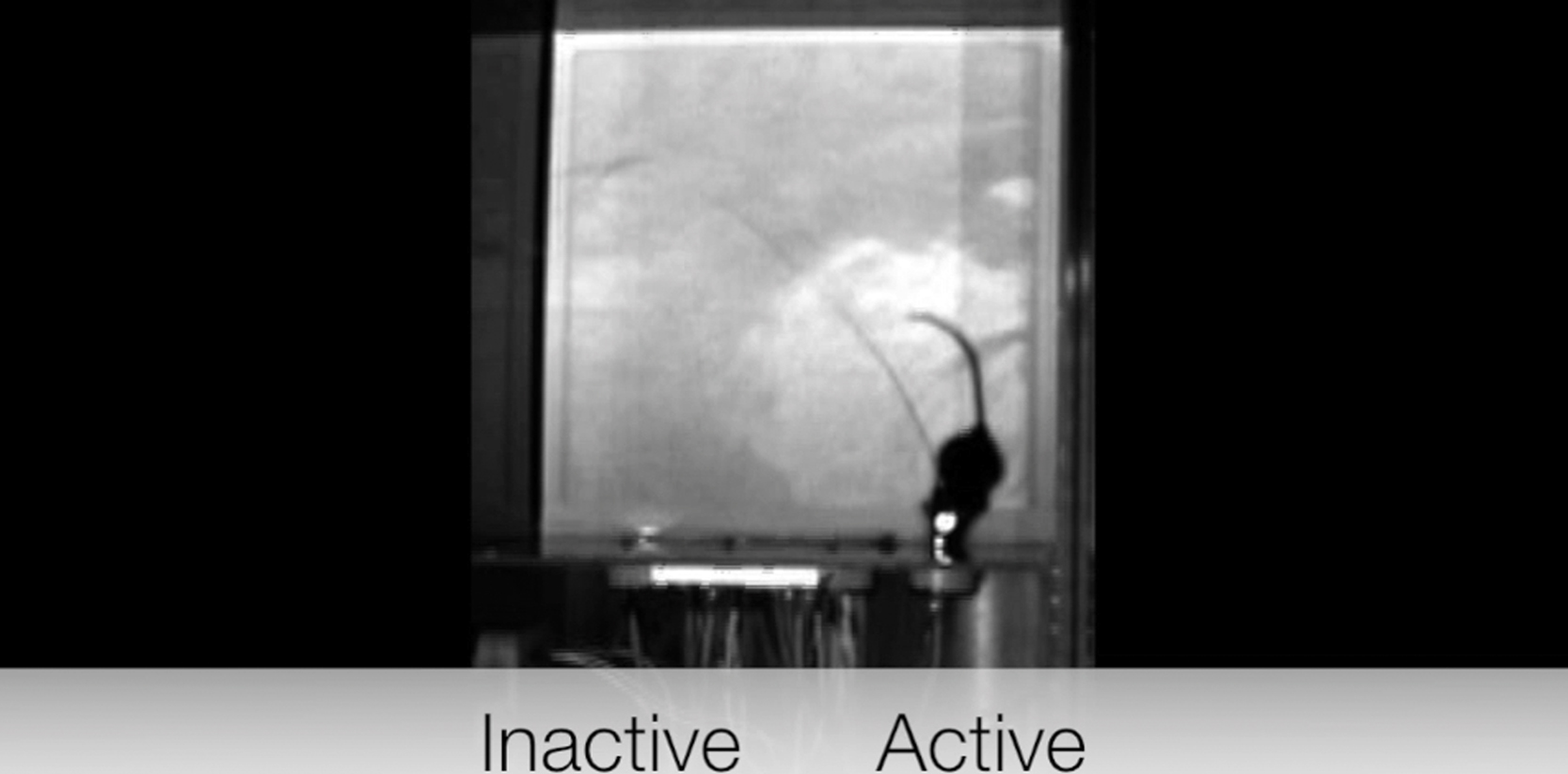

Supplement: Video 1 — Video of animal nose poking for self-stimulation. [file mmc5.jpg]
